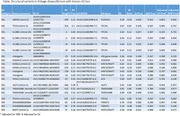# The role of common structural variants in Alzheimer’s disease

**DOI:** 10.1002/alz.092501

**Published:** 2025-01-03

**Authors:** Songmi Lee, Rui Xia, Adam C English, Gina M. Peloso, Josh Bis, Honghuang Lin, Seung Hoan Choi, Nancy Heard‐Costa, Anita L. DeStefano, Fritz J Sedlazeck, Myriam Fornage

**Affiliations:** ^1^ University of Texas Health Science Center at Houston, Houston, TX USA; ^2^ Baylor College of Medicine Human Genome Sequencing Center, Houston, TX USA; ^3^ Boston University School of Public Health, Boston, MA USA; ^4^ University of Washington, Seattle, WA USA; ^5^ University of Massachusetts Medical School, Worcester, MA USA

## Abstract

**Background:**

Structural variants (SVs), genomic alterations exceeding 50 base‐pairs, are known for their significant impact on disease pathology. However, the role of SVs in Alzheimer’s Disease (AD) remains unclear. Using a novel high‐accuracy SV calling pipeline, we analyzed a diverse sample from the Alzheimer’s Disease Sequencing Project (ADSP). Our study investigated the role of SVs in known AD loci, providing insights into the genetic architecture of AD.

**Methods:**

We included samples from ADSP 17K whole genome sequence data. SV discovery was performed using Biograph SV caller that leverages an assembly‐based method and graph‐based representation. After quality filtering at the sample‐ and variant‐ levels, there were 68,831 segmented deletions, 25,281 insertions, and 198 inversions with minor allele frequency > 1% in 11,890 individuals (5,585 cases and 6,305 controls). We focused on common SVs in linkage disequilibrium (LD) (R^2^ > 0.2) with 124 SNPs tagging known AD loci derived from previous genome‐wide association studies of AD. LD was calculated using PLINK 1.9. Association analyses of AD status were performed for each identified SV adjusting for sex, technical covariates, associated principal components, and familial relatedness using GENESIS.

**Results:**

We identified 15 deletions and 3 insertions in LD with 12 SNPs tagging known AD loci (Table). Of these, 9 SVs and 8 tagging SNPs showed nominal associations with AD (P<0.10) in the ADSP sample. The strongest AD associations were with two deletions located in an intron of *KANSL1*, in moderate LD (R^2^ = 0.65) with a SNP tagging the *MAPT* haplotype, which is known to associate with multiple neurodegenerative diseases including AD. Several SVs were in high LD with SNPs tagging AD loci. Notably, a 319‐bp deletion (R^2^ = 0.97) in the *TPCN1* locus overlapped fully with a known 309‐bp deletion known to be associated with Lewy Body dementia. A 322‐bp Alu deletion in exon 8 of *TMEM106B*, previously identified through long‐read sequencing, also demonstrated strong LD with two AD‐related SNPs (R^2^ = 0.91 and 0.89, respectively).

**Conclusion:**

Using a novel SV calling pipeline, we identified multiple AD‐related SVs, which provides additional insights into the genetic architecture of AD. Integration of omics data is warranted to explore the functional impact of these SVs.